# Estrogen-like Activity of *Scrophularia buergeriana* Root Extracts in MCF-7 Cells

**DOI:** 10.3390/biomedicines13092151

**Published:** 2025-09-04

**Authors:** Hye-Yeong Song, Jinsu Choi, Eunwoo Jeong, Harang Park, Juyeong Moon, Min-ah Kim, Javokhir Rustamov, Hwan-Soo Yoo, Tack-Joong Kim

**Affiliations:** 1 Division of Biological Science and Technology, Yonsei University, Wonju 26493, Republic of Korea; shy9987@naver.com (H.-Y.S.); wlstnbnm@naver.com (J.C.); jew0108@naver.com (E.J.); phraa@naver.com (H.P.); mjy@yonsei.ac.kr (J.M.); mina1218@yonsei.ac.kr (M.-a.K.); 2 College of Pharmacy, Chungbuk National University, Cheongju 28160, Republic of Korea; r.javohir.96.25@gmail.com (J.R.); yoohs@chungbuk.ac.kr (H.-S.Y.); 3 Research & Development Center, Doctor TJ Co., Ltd., Wonju 26494, Republic of Korea

**Keywords:** MCF-7 cells, menopause, hormone replacement therapy, phytoestrogen

## Abstract

**Background/Objectives**: Estrogen deficiency-related menopause is associated with various physical and psychological symptoms. Although hormone replacement therapy (HRT) effectively alleviates these symptoms, its long-term use is associated with several side effects such as an increased risk of breast cancer and cardiovascular disease. Consequently, there is a growing interest in some plant-derived phytoestrogens that are considered safer alternatives to estrogen. Recent studies on *Scrophularia buergeriana* confirmed their anti-inflammatory and antioxidant properties; however, their effects on menopausal health remain unclear. Therefore, the aim of this study was to investigate the estrogen-like effects of *S. buergeriana* root (SB-R) extract, a potential phytoestrogen. **Methods**: Briefly, the MCF-7 cell line, a widely used in vitro model for assessing estrogen-like activity, was treated with SB-R extract and 17β-estradiol (E2; positive control) in the presence or absence of ICI 182,780 (Fulvestrant), an estrogen receptor antagonist. An E-screen assay and flow cytometry were performed to assess the effects of the treatments on cell proliferation and the cell cycle, respectively. Additionally, Western blotting and immunofluorescence assays were performed to elucidate the potential mechanisms underlying the estrogen-like effects of SB-R. **Result**: Treatment with SB-R extract promoted MCF-7 cell proliferation in a manner similar to E2. However, ICI 182,780 co-treatment inhibited the SB-R extract-induced increase in MCF-7 cell proliferation. Additionally, SB-R extract promoted cell cycle progression by increasing the proportion of cells in the S and G2/M phases. Moreover, Western blotting and immunofluorescence assays showed that SB-R extract increased the expression of estrogen receptor alpha (ERα). Furthermore, SB-R treatment activated downstream signaling pathways by enhancing AKT and ERK phosphorylation and upregulated the expression of cell cycle regulators, including cyclin D1, cyclin dependent kinase 4 (CDK4), cyclin E1, and cyclin dependent kinase 2 (CDK2). **Conclusions**: SB-R exhibits estrogen-like activity by activating ERα-mediated AKT and ERK pathways and thereby increasing the expression of proteins involved in cell cycle regulation. This makes it a promising phytoestrogen candidate and a safer alternative to conventional hormonal therapy for alleviating menopausal symptoms.

## 1. Introduction

Menopause is a period characterized by the physiological aging of the female body, and is typically based on the cessation of menstruation for a duration of 12 months [1]. In 1990, there were 467 million postmenopausal women worldwide. This number is projected to increase to approximately 1.2 billion by 2030, with an estimated annual increase of 47 million women [2]. The average life expectancy for women is approximately 80 years, with the average age of menopause occurring around 50 years. Consequently, women generally spend about 30 years of their lives in the postmenopausal state [1,3]. Menopause is characterized by the cessation of reproductive function and is associated with various physical and psychological symptoms.

Importantly, these changes are mainly attributed to a decline in estrogen levels. Estrogen plays a crucial systemic role in the body, including maintaining bone health and central nervous system function, protecting against cardiovascular diseases, and delaying skin aging [4]. Research findings indicate that the decline in estrogen levels during menopause leads to several associated symptoms such as depression, vasomotor symptoms, hot flashes, and osteoporosis. Additionally, these symptoms present significant health challenges to women [3]. Notably, estrogen deficiency has been reported to significantly accelerate cardiovascular risk [5]. Therefore, hormone therapy, which often involves estrogen replacement, has been widely used as a primary treatment strategy to alleviate menopausal symptoms and support overall health in postmenopausal women. However, the Women’s Health Initiative (WHI) study showed that long-term hormone therapy is associated with an increased risk of breast cancer and cardiovascular events. This discovery led to a decrease in prescriptions from doctors, with many women stopping their treatment [6,7]. Accordingly, the amount of research on safer natural estrogen alternatives has increased, with phytoestrogens attracting attention as a potential substitute.

Phytoestrogens are plant-derived compounds that structurally mimic mammalian estrogens and are capable of binding to estrogen receptors [8,9,10,11]. Phytoestrogens, including isoflavones, lignans, coumestans, and flavonoids, are naturally present in various foods such as legumes, grains, and fruits [9]. Considering their unique characteristics, scientists are actively studying the estrogen-like activities of phytoestrogens, which has resulted in the discovery of novel materials [12,13]. However, some existing phytoestrogens have low biological activity and can cause side effects such as liver toxicity, which indicates the need for further research on safer and more effective phytoestrogens [14,15].

*Scrophularia buergeriana* belongs to the family Scrophulariaceae and is native to Korea and China. It has traditionally been used as a medicinal plant to treat fever [16]. Notably, the main components identified in the root of *S. buergeriana* include harpagoside, angoroside C, aucubin, and acetoside [17]. Research findings indicate that these compounds possess several pharmacological effects, including neuroprotective, antioxidant, and anti-inflammatory properties [18,19,20,21]. However, most existing research on *S. buergeriana* has focused on mechanisms related to inflammation and oxidative stress, with there being limited studies on its effects on menopause-associated symptoms. Therefore, the aim of this study was to explore new pharmacological functions of *S. buergeriana* and evaluate its estrogen-like activity to improve menopausal health in women.

## 2. Materials and Methods

### 2.1. Preparation of S. buergeriana Root Extracts

*S. buergeriana* root (SB-R) extract was provided by the Rural Development Administration (RDA, Jeonju-si, Republic of Korea). *Scrophularia buergeriana* was cultivated by the Good Agricultural Practice (GAP) of the Rural Development Administration (RDA) and harvested in Eumseong, Korea (GPS: E 128°62′ N 36°56′). For sample preparation, the dried *S. buergeriana* was extracted three times with 2 L of water at 100 °C for one day. The resulting extracts were filtered through Whatman No. 1 paper, combined, and concentrated using a rotary evaporator (EYELA N-1000, Tokyo, Japan) at 40 °C. The concentrated extract was stored in the form of dried powder for use in experiments. SB-R was dissolved in dimethyl sulfoxide (DMSO) at various concentrations.

### 2.2. Chemical Reagent

17β-estradiol (E2; Sigma-Aldrich, St. Louis, MO, USA) was used as a positive control to evaluate estrogen-like activity in MCF-7 cells. ICI 182,780 (Fulvestrant; Santa Cruz Biotechnology, Dallas, TX, USA) was used as an estrogen receptor antagonist.

### 2.3. In Silico Estrogen Receptor α Molecular Docking

An in silico molecular docking simulation was performed on the ligand binding domain of estrogen receptor alpha (ERα). Briefly, the protein structure of ERα was downloaded from the Protein Data Bank (PDB) with PDB ID 1 × 7R.

Four active compounds from *S. buergeriana* (harpagoside, angoroside, acetoside, and aucubin) were selected based on a previous study [17]. Additionally, the chemical structures of the compounds were downloaded from the PubChem database. Ligand structures were converted to 3D structures, energy minimization was performed using Avogadro 1.2.0, and then the structures were converted to PDB files using Open Babel 2.4.1 [22]. Chains other than chain A and non-standard residues (including genistein) were removed from the ERα structure using University of California San Francisco (UCSF) Chimera 1.19 [23]. Thereafter, ligand and receptor files were prepared using the Assisted Molecular Docking (AMDock) 1.5.2 interface [24]. Molecular docking was performed using the AutoDock Vina 1.2.1 protocol [25]. Visualization of the molecular modeling results and hydrogen bond analysis were performed using UCSF Chimera 1.19 [23].

### 2.4. Cell Culture

The estrogen receptor-positive human breast cancer cell line MCF-7 was purchased from the American Type Culture Collection (Rockville, MD, USA). Briefly, MCF-7 was cultured in Dulbecco’s modified Eagle’s medium (DMEM; Gibco, Grand Island, NY, USA) containing 10% fetal bovine serum (FBS; Access Biologicals, Vista, CA, USA) and penicillin/streptomycin solution (100 U/mL penicillin, 100 μg/mL streptomycin; Gibco, Grand Island, NY, USA). Cell cultures were maintained at 37 °C in a humidified atmosphere containing 5% CO_2_. For the E-screen assay, Western blotting, cell cycle analysis, and immunofluorescence assay, MCF-7 cells were cultured in estrogen- and phenol red-free DMEM (Gibco, Grand Island, NY, USA) containing 5% charcoal-stripped fetal bovine serum (CS-FBS; Gibco, Grand Island, NY, USA) and penicillin/streptomycin solution [26]. For all experiments, the negative control group was treated with the vehicle (DMSO) only, under the same culture conditions.

### 2.5. Estrogen-like Activity of SB-R in MCF-7 Cells (E-Screen Assay)

MCF-7 cells were seeded in a 24-well plate (1 × 10^5^ cells per well) in DMEM containing 10% FBS and penicillin/streptomycin solution for 24 h. Thereafter, the medium was replaced with phenol red-free DMEM containing 5% CS-FBS and various concentrations (25, 50, 100, and 200 μg/mL) of SB-R extract or E2 [27]. For the antagonistic test, MCF-7 cells were treated with the estrogen receptor antagonist ICI 182,780 (Fulvestrant; Santa Cruz Biotechnology) for 144 h [28,29]. Thereafter, 100 μL of 3-(4,5-Dimethylthiazol-2-yl)-2,5-diphenyl tetrazolium bromide (MTT solution; Duchefa Biochemie, Haarlem, The Netherlands) was added to each well. After 2 h of incubation, the formazan crystals were dissolved with DMSO [30], and the absorbance was measured at 595 nm using a plate reader.

### 2.6. Cell Cycle Analysis

Briefly, MCF-7 cells were seeded in a 6-well plate (2 × 10^5^ cells per well) in DMEM containing 10% FBS and penicillin/streptomycin solution for 24 h. After washing with phosphate-buffered saline (PBS, pH 7.4; Gibco, Grand Island, NY, USA), the cells were treated with various concentrations (100 and 200 μg/mL) of SB-R extract or E2 in a medium containing 5% CS-FBS in the presence or absence of the estrogen receptor antagonist ICI 182,780 for 24 h. Cells were detached from each well using trypsin–ethylenediaminetetraacetic acid (trypsin–EDTA; Sigma-Aldrich, St. Louis, MO, USA). After adding 2% serum, the cells were centrifuged at 1000× *g* for 5 min to remove the supernatant. Thereafter, the pellet was gently resuspended in PBS for washing and centrifuged at 1000× *g* for 5 min. After discarding the supernatant, cold 70% ethanol was added dropwise to the pellet under gentle vortexing [31]. After fixing at −20 °C or 4 °C overnight, the cells were centrifuged at 5000 rpm for 5 min and washed twice with PBS to remove ethanol. RNase A was added for RNA degradation and the mixture was incubated at room temperature (21 ± 3 °C) for 30 min. The cells were transferred to round tubes, stained with propidium iodide (PI) solution, and incubated in the dark at room temperature for 10 min. Cell cycle analysis was performed on the BD FACSCalibur™ Flow Cytometer (BD Biosciences, San Jose, CA, USA).

### 2.7. Western Blot Analysis

For Western blotting, MCF-7 cells were treated and cultured under the same conditions as those used for cell cycle analysis (Section 2.6). After washing with PBS (pH, 7.4), the cells were lysed with the PRO-PREP protein extraction solution (iNtRON Biotechnology, Seongnam-si, Republic of Korea) to extract the total protein. Thereafter, the extracted protein was quantified via a protein assay dye reagent concentrate (Bio-Rad, Hercules, CA, USA) using the Bradford method, with bovine serum albumin (BSA) used as the standard. Absorbance was measured at 595 nm using a plate reader. Equal amounts of protein were mixed with sodium dodecyl sulfate (SDS) sample buffer and boiled at 100 °C for 10 min. Thereafter, the proteins were separated using 8% SDS-polyacrylamide gel electrophoresis and transferred into an immunoblotting polyvinylidene fluoride membrane (Bio-Rad, Hercules, CA, USA) and nitrocellulose membrane (Bio-Rad, Hercules, CA, USA) in a wet transfer system. After blocking with a blocking reagent, the membrane was incubated overnight at 4 °C with specific primary antibodies (1:2500) that target ERα (Cell Signaling Technology, Danvers, MA, USA; #8644), p-AKT (Cell Signaling Technology, Danvers, MA, USA; #9271), AKT (Cell Signaling Technology, Danvers, MA, USA; #9272), p-ERK (Cell Signaling Technology, Danvers, MA, USA; #4377), ERK (Cell Signaling Technology, Danvers, MA, USA; #4695), cyclin E1 (Cell Signaling Technology, Danvers, MA, USA; #4129), cyclin D1 (Cell Signaling Technology, Danvers, MA, USA; #2978), cyclin-dependent kinase 2 (CDK2; Cell Signaling Technology, Danvers, MA, USA; #2546), CDK4 (Cell Signaling Technology, Danvers, MA, USA; #12790), and glyceraldehyde-3-phosphate dehydrogenase (GAPDH; Cell Signaling Technology, Danvers, MA, USA; #5174). Thereafter, the cells were incubated with a secondary antibody (1:5000) at room temperature for 2 h. Protein bands were visualized on ChemiDoc™ XRS+ Imaging System (Bio-Rad, Hercules, CA, USA) using WestGlow™ PICO PLUS chemiluminescent substrate (Biomax, Guri-si, Korea) enhanced chemiluminescence solution.

### 2.8. Immunofluorescence Analysis

Briefly, MCF-7 cells were seeded in a 6-well plate (2 × 10^5^ cells/well) on coverslips for 24 h. Thereafter, the cells were treated with various concentrations (100 and 200 μg/mL) of SB-R extract or E2 for 24 h. After two washes with PBS, the cells were treated with 4% formaldehyde solution in PBS at room temperature for 10 min, washed three times with PBS, and permeabilized with 0.2% Triton X-100 in PBS for 20 min on ice [32]. Subsequently, the cells were washed three times with PBS and treated with 3% BSA in PBS at room temperature for 30 min. After blocking, the cells were incubated with primary ERα antibody (Thermo Fisher Scientific, Waltham, MA, USA; # MA1-310) at room temperature for 2 h or at 4 °C overnight. Thereafter, the cells were incubated with highly cross-adsorbed Alexa Fluor 594-conjugated goat anti-mouse IgG (H  +  L) (Thermo Fisher Scientific, Waltham, MA, USA) at room temperature for 1 h. Finally, the cells were stained with 4′,6-diamino-2-phenylindole (DAPI) mounting solution, which was dropped onto a slide glass, and the coverslip was placed directly on top of the extraction solution with the cells facing down [33]. Imaging was performed using a fluorescence microscope (Olympus, Tokyo, Japan).

### 2.9. Statistical Analysis

All statistical analyses were performed using GraphPad Prism 5 (GraphPad Software Inc.; Boston, MA, USA). Data are presented as mean ± standard error of the mean (SEM). Significant differences between groups were determined using *t*-tests and one-way analysis of variance (ANOVA), which was determined with Dunnett’s multiple comparison test. Statistical significance was set at *p* < 0.05.

## 3. Results

### 3.1. Estrogen Receptor α Molecular Docking

**Figure 1:**
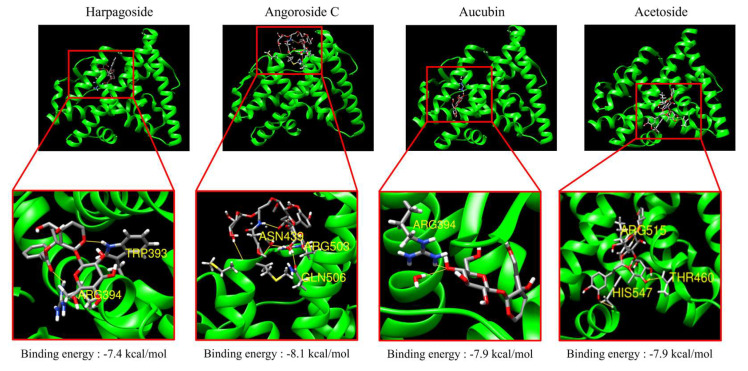
Estrogen receptor α molecular docking. Docking poses were generated via molecular docking simulations using AutoDock Vina in the AMDock interface. The binding sites of each compound were visualized in enlarged views, and the amino acid residues involved in hydrogen bonding were identified using UCSF Chimera 1.19. Yellow lines represent putative hydrogen bonds. Specific hydrogen bonds were identified: harpagoside formed hydrogen bonds with Trp393 and Arg394; angoroside C with Asn439, Arg503, and Gln506; aucubin with Arg394; and acetoside with His547, Thr460, and Arg515.

A molecular docking study was conducted to evaluate the binding affinity of four active compounds in SB-R (harpagoside, angoroside C, aucubin, and acetoside) for ERα. Notably, the binding energies of harpagoside, angoroside C, aucubin, and acetoside were −7.4, −8.1, −7.9, and −7.9 kcal/mol, respectively, which indicates that all four compounds possessed a favorable binding affinity for Erα (Figure 1). Overall, these results provide evidence of their potential for effective interaction with ERα.

### 3.2. Estrogen-like Activity of SB-R in the E-Screen Assay

**Figure 2:**
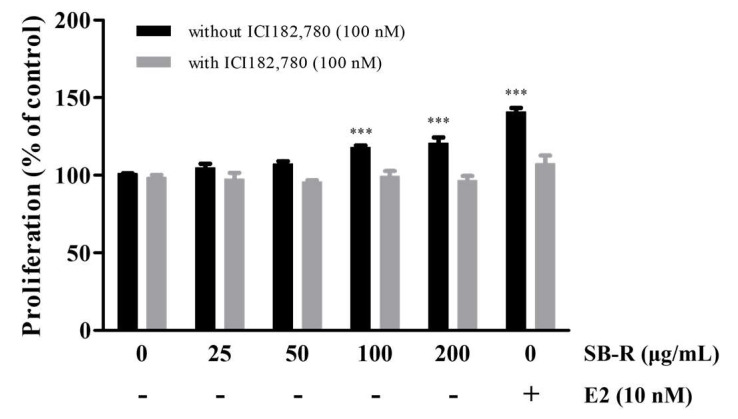
Estrogen-like activity of *S. buergeriana* root (SB-R) extract in the E-screen assay. MCF-7 cells were treated with varying concentrations of SB-R (25, 50, 100, and 200 μg/mL) and E2 (10 nM) for 144 h. To test for antagonistic effects, the ER antagonist ICI 182,780 (100 nM) was added to the samples. MCF-7 cells were analyzed using MTT assay. The optical density (OD) was measured at 595 nm. Data are presented as mean ± standard error of mean (SEM; n = 3). *** *p* < 0.001 compared with control group.

To examine whether the SB-R extract exhibited estrogen-like activity through cell proliferation, an E-screen assay was performed. Treatment with 100 and 200 µg/mL of SB-R extract and 10 nM of E2 significantly increased the cell proliferation to 115 ± 1.24, 122 ± 3.8, and 139 ± 2.7%, respectively. However, co-treatment with the estrogen receptor antagonist ICI 182,780 (100 nM) inhibited cell proliferation (Figure 2).

### 3.3. Effects of SB-R on Cell Cycle Progression

**Figure 3:**
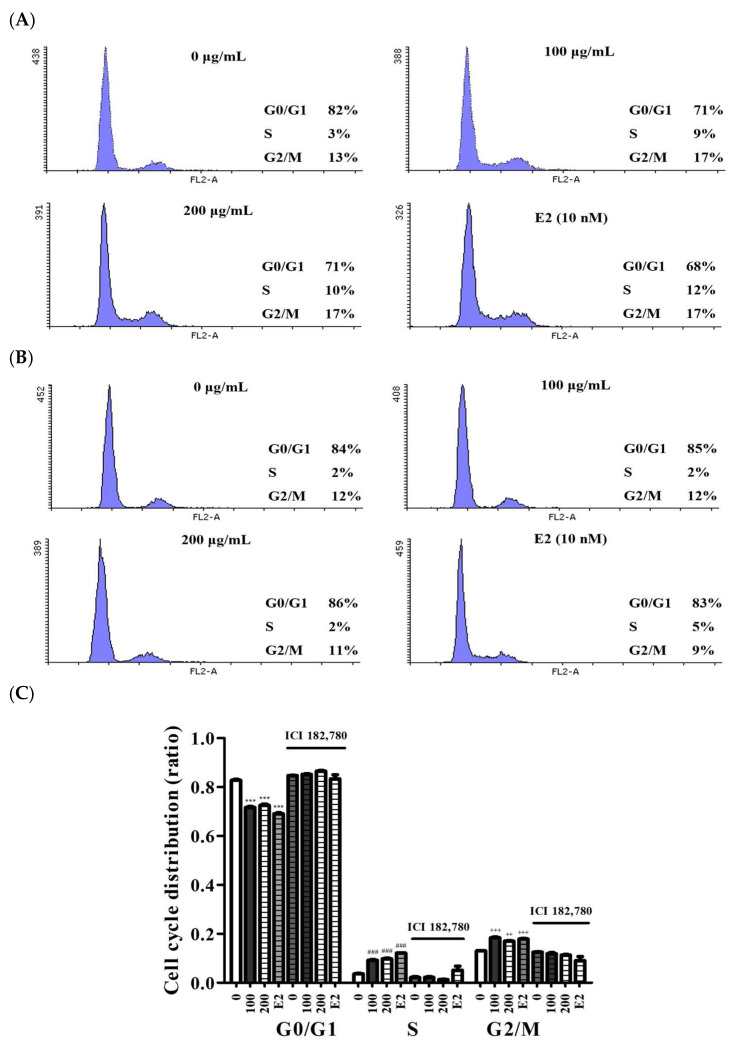
Effects of *S. buergeriana* root (SB-R) extract on cell cycle progression. (**A**) MCF-7 cells were treated with SB-R (100 and 200 µg/mL) and E2 in the absence of ICI 182,780 (100 nM). (**B**) MCF-7 cells were treated with SB-R extract (100 and 200 µg/mL) and E2 in the presence ICI 182,780 (100 nM). (**C**) Cell cycle distribution was assessed using propidium iodide (PI) staining and flow cytometry. Percentages of cells in the G0/G1, S, and G2/M phases of the cell cycle. Data are presented as mean ± standard error of mean (SEM; n = 3). *** *p* < 0.001 compared with the G0/G1 control group; ### *p* < 0.001 compared with the S phase control group; ++ *p* < 0.01, +++ *p* < 0.001 compared with the G2/M control group.

Flow cytometry was performed to examine the effects of SB-R extract on cell cycle phases. MCF-7 cells were treated with SB-R extract (100 and 200 µg/mL) and E2 (10 nM). The SB-R extract and E2 treatments caused a significant decrease in the proportion of cells in the G0/G1 phase, reducing it from 82% in the control group to 71% in the SB-R extract (100 and 200 µg/mL) and 68% in the E2. Concurrently, the proportion of cells in the S phase increased from 3% in the control group to 9% and 10% in the SB-R extract (100 and 200 µg/mL) and to 12% in the E2. Similarly, the G2/M phase proportion increased from 13% in the control group to 17% in the SB-R extract (100 and 200 µg/mL) and E2. However, co-treatment with 100 nM of ICI 182,780 inhibited the SB-R extract- and E2-induced decreases in the G0/G1 phase and increases in the S and G2/M phases. Specifically, with 100 and 200 µg/mL of SB-R extract and 10 nM of E2, the proportion of cells in the G0/G1 phase remained at 85%, 86%, and 83%, respectively. Concurrently, the proportion of cells in the S phase decreased from 9%, 10%, and 12% to 2%, 2%, and 5%, respectively, and the proportion of cells in the G2/M phase decreased from 17%, 17%, and 17% to 12%, 11%, and 9%, respectively (Figure 3).

### 3.4. Effect of SB-R on ERα Expression

**Figure 4:**
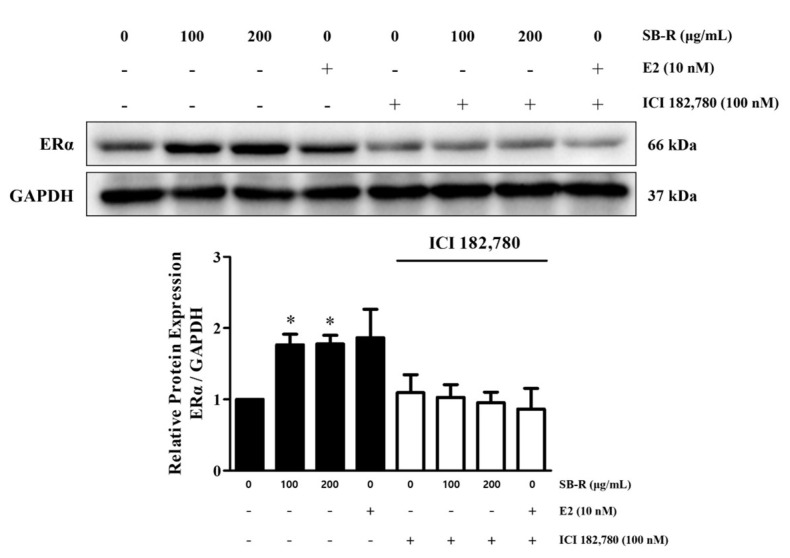
Effect of *S. buergeriana* root (SB-R) extract on ERα expression. MCF-7 cells were treated with SB-R (100 and 200 µg/mL) and E2 (10 nM) in the absence or presence ICI 182,780 (100 nM) for 24 h. ERα and GAPDH protein levels in MCF-7 cells. Data are presented as mean ± standard error of mean (SEM; n = 3). * *p* < 0.05 compared with the control group.

To determine whether SB-R extract exerts its effects via the estrogen receptor pathway, the ERα protein expression was analyzed. The expression of ERα has been shown to increase following binding with estrogen or similar structures. MCF-7 cells were treated with SB-R extract (100 and 200 µg/mL) or E2 (10 nM). The SB-R extract and E2 treatments significantly increased the ERα expression in the cells. The relative densities were increased by 1.77 ± 0.15-, 1.78 ± 0.12-, and 1.87 ± 0.40-fold in protein treated with 100 and 200 µg/mL of SB-R extract and 10 nM of E2, respectively. However, co-treatment with the estrogen receptor antagonist ICI 182,780 (100 nM) inhibited SB-R extract- and E2-induced increases in ERα expression (Figure 4).

### 3.5. Immunofluorescence Analysis of ERα Expression

**Figure 5:**
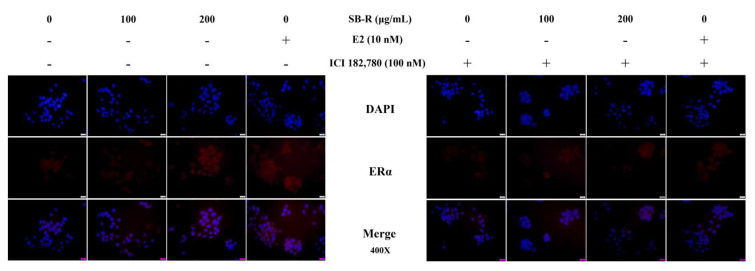
Immunofluorescence analysis of ERα expression. MCF-7 cells were treated with *S. buergeriana* root (SB-R) extract (100 and 200 µg/mL) and 17β-estradiol (E2; 10 nM) in the presence or absence of ICI 182,780 (100 nM) for 24 h. ERα expression in the cytoplasm was stained red using Alexa Fluor 594. Nuclei were stained blue using DAPI. Images were captured at ×400 magnification using a fluorescence microscope.

An immunofluorescence assay was performed to visually observe the changes in ERα expression in MCF-7 cells following their treatment with SB-R extract (100 and 200 µg/mL) and E2 (10 nM). Treatment with SB-R extract and E2 caused a dose-dependent increase in the ERα signal in the cells. However, co-treatment with 100 nM of ICI 182,780 inhibited the SB-R extract- and E2-induced increases in the ERα signal (Figure 5).

### 3.6. Effects of SB-R on AKT and ERK Phosphorylation

**Figure 6:**
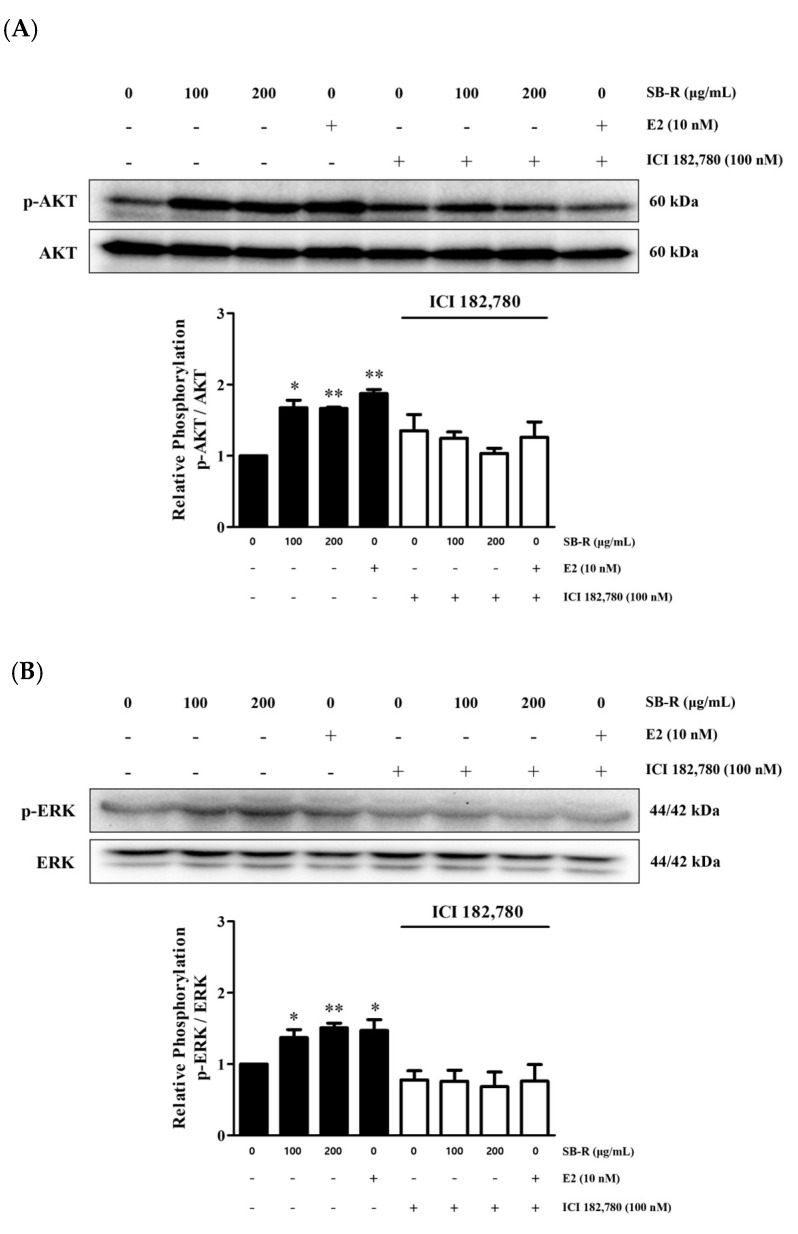
Effects of *S. buergeriana* root (SB-R) extract on AKT and ERK phosphorylation. MCF-7 cells were treated with SB-R (100 and 200 µg/mL) and 17β-estradiol (E2; 10 nM) in the absence or presence of ICI 182,780 (100 nM) for 24 h. Phosphorylation levels of (**A**) AKT and (**B**) ERK in MCF-7 cells. Data are presented as mean ± standard error of mean (SEM; n = 3). * *p* < 0.05, ** *p* < 0.01 compared with control group.

Western blot analysis was performed to examine the phosphorylation levels of AKT and ERK, key signaling proteins downstream of estrogen receptor activation, to investigate the pathways involved in the SB-R extract-induced increase in cell proliferation. MCF-7 cells were treated with SB-R extract (100 and 200) and E2 (10 nM). The SB-R extract and E2 treatments significantly increased the AKT and ERK phosphorylation levels in the cells. The relative densities of phosphorylated AKT were increased by 1.68 ± 0.10-, 1.67 ± 0.02-, and 1.87 ± 0.06-fold in cells treated with 100 and 200 µg/mL of SB-R extract and 10 nM of E2, respectively. Similarly, the relative densities of phosphorylated ERK were increased by 1.37 ± 0.11-, 1.50 ± 0.07-, and 1.47 ± 0.15-fold in cells treated with 100 and 200 µg/mL of SB-R extract and 10 nM of E2, respectively. However, ICI 182,780 co-treatment (100 nM) inhibited the SB-R extract- and E2-induced increases in AKT and ERK phosphorylation (Figure 6).

### 3.7. Effects of SB-R on Cyclin D1 and CDK4 Expression

**Figure 7:**
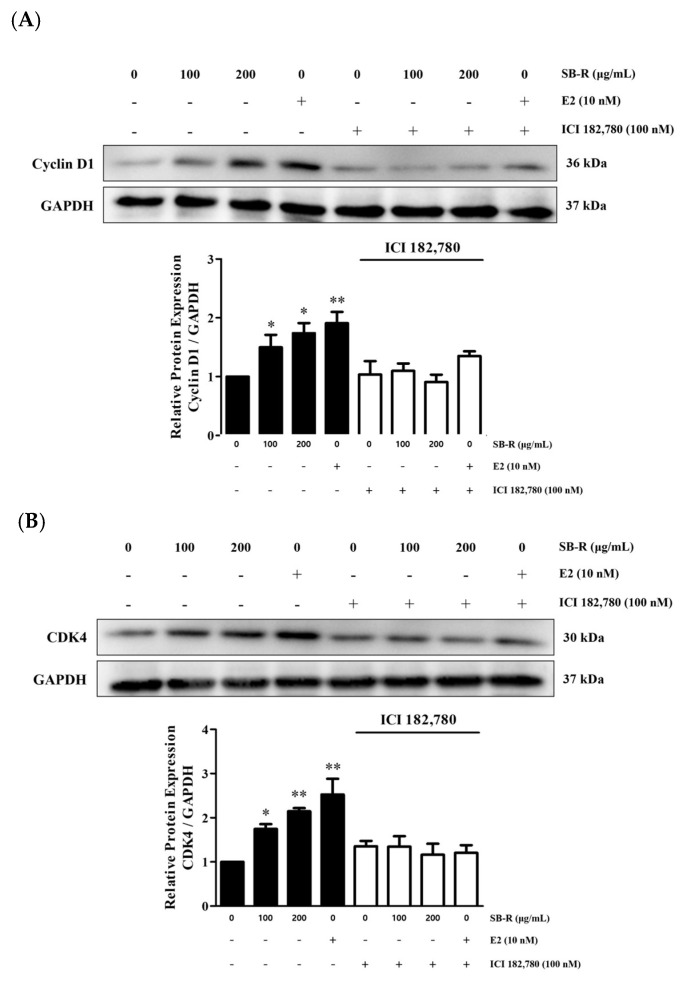
Effects of *S. buergeriana* root (SB-R) extract on the expression of cyclin D1 and CDK4. MCF-7 cells were treated with SB-R (100 and 200 µg/mL) and 17β-estradiol (E2; 10 nM) in the absence or presence of ICI 182,780 (100 nM) for 24 h. (**A**) Cyclin D1 and (**B**) CDK4 expression levels in MCF-7 cells. GAPDH was used as the loading control. Data are presented as mean ± standard error of mean (SEM; n = 3). * *p* < 0.05, ** *p* < 0.01 compared with control group.

Western blot analysis was performed to examine the changes in the expression of molecules involved in cell cycle regulation, including cyclin D1 and CDK4. MCF-7 cells were treated with SB-R extract (100 and 200 µg/mL) and E2 (10 nM). The SB-R extract and E2 treatments significantly increased the cyclin D1 and CDK4 expression levels compared with those in the untreated group. The relative densities of cyclin D1 were increased by 1.50 ± 0.21-, 1.74 ± 0.17-, and 1.90 ± 0.19-fold in cells treated with 100 and 200 µg/mL of SB-R extract and 10 nM of E2, respectively. Similarly, the relative densities of CDK4 were increased by 1.74 ± 0.11-, 2.14 ± 0.07-, and 2.52 ± 0.36-fold in cells treated with 100 and 200 µg/mL of SB-R extract and 10 nM of E2, respectively. However, ICI 182,780 co-treatment (100 nM) inhibited the SB-R extract- and E2-induced increases in the expression of the molecules (Figure 7).

### 3.8. Effects of SB-R on the Expression of Cyclin E1 and CDK2

**Figure 8:**
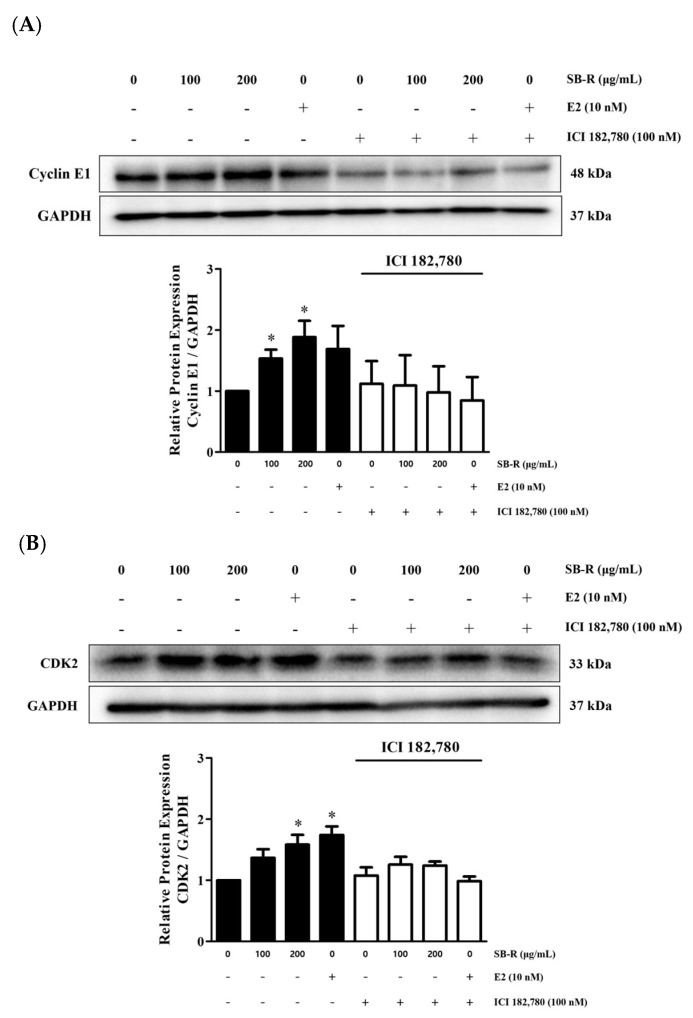
Effects of *S. buergeriana* root (SB-R) on the expression of cyclin E1 and CDK2. MCF-7 cells were treated with SB-R (100 and 200 µg/mL) and 17β-estradiol (E2; 10 nM) in the absence or presence of ICI 182,780 (100 nM) for 24 h. Protein expression levels of (**A**) cyclin E1 and (**B**) CDK2 in MCF-7 cells. GAPDH was assessed as the loading control. Data for cyclin E1 are presented as mean ± standard error of mean (SEM; n = 4), and data for CDK2 are presented as mean ± standard error of mean (SEM; n = 3). * *p* < 0.05 compared with the control group.

Western blot analysis was performed to examine the changes in the expression of molecules involved in cell cycle regulation, including cyclin E1 and CDK2. CDK2 is involved in the G1/S transition and forms a complex with cyclin E1 to induce cell entry into the S phase. MCF-7 cells were treated with SB-R extract (100 and 200 µg/mL) and E2 (10 nM) in the presence or absence of ICI 182,780 (100 nM). Treatment with SB-R extract and E2 significantly increased the cyclin E1 and CDK2 expression levels compared with those in the untreated group. The relative densities of cyclin E1 were increased by 1.53 ± 0.14-, 1.88 ± 0.27-, and 1.69 ± 0.38-fold in cells treated with 100 and 200 µg/mL of SB-R extract and 10 nM of E2, respectively. Similarly, the relative densities of CDK2 were increased by 1.37 ± 0.14-, 1.58 ± 0.16-, and 1.74 ± 0.14-fold in cells treated with 100 and 200 µg/mL of SB-R extract and 10 nM of E2, respectively. However, co-treatment with 100 nM of ICI 182,780 inhibited the SB-R extract- and E2- induced upregulation of the molecules (Figure 8).

## 4. Discussion

Menopause is accompanied by the loss of reproductive capacity and various physical and psychological symptoms [4]. Phytoestrogens are natural estrogen alternatives with fewer side effects. Recently, these compounds have attracted increasing attention as potential therapeutic agents [10]. Previous studies on *S. buergeriana* extract have primarily focused on its anti-inflammatory and antioxidant properties [19], with there being limited information on its role in menopausal health in women. In this study, we found that SB-R extract exhibited estrogen-like activity in MCF-7 cells. MCF-7 cells are a human breast cancer cell line characterized by high expression of estrogen receptors [34]. Additionally, MCF-7 cells exhibit a high responsiveness to estrogenic substances and are widely used in E-screen assays and estrogen pathway studies. Moreover, the proliferation of MCF-7 cells is highly responsive to estrogen receptor activation, providing a suitable model for assessing the estrogen-like activity of substances [29].

Considering that 10 nM of the endogenous estrogen E2 has been shown to effectively activate estrogen receptors and promote MCF-7 cell proliferation [35], and that it is widely used as a standard dose in screening assays, MCF-7 cells were treated with 10 nM of E2 (positive control) in the present study. ICI 182,780 (fulvestrant), an estrogen receptor-degrading antagonist, was used at a concentration of 100 nM [36]. Notably, this concentration can block the ERα pathway in MCF-7 cells. Additionally, several studies have shown that similar concentrations inhibit estrogen signaling [37,38]. Therefore, MCF-7 cells were co-treated with ICI 182,780 to confirm whether SB-R acts via the estrogen receptor. Considering that low levels of steroid hormones, especially estrogen, can remain in phenol red medium and FBS [27], phenol red-free DMEM and 5% CS-FBS were used in this study to prevent any interference from endogenous estrogen in the culture medium [39].

Molecular docking is an in silico analytical method for predicting the binding potential between ligands and receptors. This allows the evaluation of the structural properties and binding affinities of bioactive compounds that interact with the active site of a receptor [40,41]. Accordingly, a molecular docking simulation was conducted to evaluate the potential binding of four compounds [17] in SB-R (harpagoside, angoroside C, aucubin, and acetoside) to ERα. Notably, the binding energies of harpagoside, angoroside C, aucubin, and acetoside were −7.4, −8.1, −7.9, and −7.9 kcal/mol, respectively (Figure 1), which suggests favorable binding to ERα [42,43,44,45]. Generally, the more negative the binding energy (i.e., the greater the absolute value), the stronger the predicted binding potential with the receptor [46]. Overall, these results show the potential of the compounds in SB-R to form stable interactions with ERα. Additionally, the docking simulation predicted that harpagoside would form hydrogen bonds with Trp393 and Arg394; angioside C with Asn439, Arg503, and Gln506; aucubin with Arg394; and acetoside with His547, Thr460, and Arg515. Particularly, some compounds were predicted to form hydrogen bonds with residues located within the classical estrogen receptor binding site, such as Arg394 and His547 (Figure 1) [47,48]. Overall, this predicted interaction with the classical site suggests the potential of the SB-R compounds to bind to ERα.

However, the accuracy of molecular docking predictions is affected by several factors, including the precision of the receptor and ligand 3D structures, the algorithms used for binding energy calculations, and inherent limitations of the scoring function. Therefore, definitively determining biological binding based solely on binding-affinity values has limitations [49]. Based on the limitations of molecular docking modeling, an E-screen assay was performed to evaluate the estrogen-like activity of SB-R in MCF-7 cells.

E-screen assay is a method that is widely used to evaluate estrogen-like activity by measuring MCF-7 cell proliferation in the presence of estrogenic compounds. SB-R extract and E2 treatments markedly increased the proliferation of MCF-7 cells compared with that in the untreated group. However, ICI 182,780 co-treatment attenuated the E2- and SB-R extract-induced increases in MCF-7 cell proliferation, which suggests that SB-R induced proliferation via the estrogen receptor pathway (Figure 2). Additionally, flow cytometry was performed to examine the effects of SB-R extract on cell cycle progression. SB-R extracts and E2 significantly decreased the proportion of cells in the G0/G1 phase and increased the proportion of cells in the S and G2/M phases (Figure 3). Overall, these results suggest that SB-R extract promotes cell cycle progression by promoting DNA synthesis and mitosis.

Western blotting and immunofluorescence assays were performed to detect ERα protein expression to confirm whether SB-R exerted estrogen-like activity via the estrogen receptor pathway. Estrogen and phytoestrogens can bind to the ligand-binding domain of ERα through non-covalent interactions such as hydrogen bonding, van der Waals forces, and π–π stacking [50,51]. Although their binding affinity is generally lower than that of E2, these substances have sufficient structural similarity to induce transcriptional activity [52]. ERα expression increases following binding with estrogen or structurally related substances. Our study showed that SB-R extract and E2 treatments increased the ERα expression in MCF-7 cells. However, ICI 182,780 co-treatment inhibited the E2- and SB-R extract-induced increases in ERα expression (Figure 4 and Figure 5), which indicates that the effect of SB-R extract was mediated by the estrogen receptor. Consistent with the results of Western blotting, the immunofluorescence assay confirmed that SB-R extract and E2 enhanced the ERα signal, whereas ICI 182,780 co-treatment inhibited the ERα signal.

AKT is a downstream signaling protein that is activated following estrogen receptor stimulation and involved in cell proliferation and transcriptional regulation [53,54,55]. SB-R extract and E2 treatments increased the AKT phosphorylation in a concentration-dependent manner. However, ICI 182,780 co-treatment abolished the increase in AKT phosphorylation, which suggests that SB-R extract increased the MCF-7 cell proliferation via ERα-AKT signaling (Figure 6A). ERK is another signaling molecule activated by estrogen. ERα acts as an upstream regulator of the MAPK pathway to promote ERK phosphorylation [56,57]. The ERK phosphorylation increased in a concentration-dependent manner following treatment with SB-R extract and E2. However, ICI 182,780 co-treatment inhibited the SB-R extract- and E2-induced increases in ERK phosphorylation (Figure 6B).

Cyclin D1 is regulated by ERα and is also influenced indirectly through the AKT and ERK signaling pathways [58]. Cyclin D1 binds to CDK4, which promotes cell cycle progression during the early G1 phase. Additionally, cyclin E1 binds to CDK2, facilitating the transition from the G1 to the S phase [59,60]. Notably, the formation of these cyclin–CDK complexes requires an increase in the expression of individual proteins to promote cell cycle entry [61].

**Figure 9:**
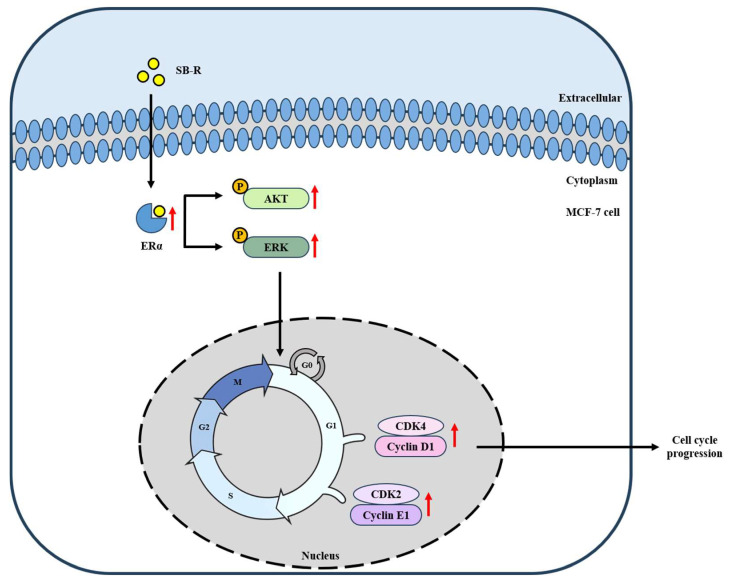
Proposed mechanism of *S. buergeriana* root (SB-R) via ERα signaling pathway in MCF-7 cells.

Considering that SB-R extract and E2 enhanced ERα expression and increased AKT and ERK phosphorylation, we examined the expression of cyclin D1, CDK4, cyclin E1, and CDK2. SB-R extract and E2 treatments upregulated the expression of these cell cycle regulators in a concentration-dependent manner. However, ICI 182,780 co-treatment inhibited the E2- and SB-R-induced increases in the regulators (Figure 7 and Figure 8), which suggests that SB-R regulated cell cycle progression through ERα-mediated signaling. Overall, these results indicate that SB-R extract promotes cell proliferation by regulating the cell cycle. This involves an increase in ERα expression, AKT and ERK phosphorylation, and formation of cyclin D1–CDK4 and cyclin E1–CDK2 complexes (Figure 9).

This study established the ERα-dependent activity of SB-R. However, the possibility of SB-R acting through other estrogen receptor subtypes, such as the estrogen receptor β (ERβ) and G-protein-coupled estrogen receptor (GPER), cannot be excluded. ERβ is associated with anti-inflammatory functions [62], and the GPER is located on the cell membrane. It has been reported to activate the PI3K/Akt and MAPK/ERK pathways through rapid non-transcriptional signaling. This activation is associated with cardiovascular protection and metabolic regulation [63]. Therefore, the possibility of SB-R acting on the ERβ and GPER cannot be ruled out, and this remains a crucial aspect to be established in future studies. In future research, we aim to comprehensively analyze the effects of SB-R on various estrogen receptor subtypes to more comprehensively clarify its molecular mechanisms.

## 5. Conclusions

Our findings suggest that SB-R extract promotes cell proliferation through an estrogen-like activity, highlighting its potential for the treatment of menopausal symptoms. Based on our findings, SB-R extract can be considered a possible alternative to hormone replacement therapy. However, although this study confirmed the estrogen-like activity of SB-R extract in MCF-7 cells, in vivo, preclinical, and clinical validations are necessary to facilitate its application in the treatment of menopausal symptoms. Furthermore, we did not assess whether SB-R extract improved specific physiological or psychological symptoms. Consequently, additional research utilizing animal models and clinical trials is required to evaluate the therapeutic benefits of SB-R in menopause-related conditions. Further, the clinical application of SB-R faces several significant challenges that necessitate further investigation. These include (i) the standardization and stringent quality control of the extract to ensure reproducible biological activity, which is crucial for botanical extracts with complex compositions; (ii) comprehensive in vivo and long-term safety evaluations designed to assess chronic effects and potential interactions within biological systems, extending beyond acute toxicity; and (iii) a thorough consideration of the multifactorial nature of menopausal symptoms, recognizing that a comprehensive therapeutic approach may necessitate combined or integrated treatment strategies rather than a single intervention. Systematically addressing these translational challenges will be pivotal for advancing SB-R from promising preclinical findings towards successful clinical application.

## Data Availability

The original contributions presented in this study are included in the article. Further inquiries can be directed to the corresponding author.
